# Brain–body-task co-adaptation can improve autonomous learning and speed of bipedal walking

**DOI:** 10.1088/1748-3190/ad8419

**Published:** 2024-10-24

**Authors:** Darío Urbina-Meléndez, Hesam Azadjou, Francisco J Valero-Cuevas

**Affiliations:** 1 Alfred E. Mann Department of Biomedical Engineering, University of Southern California, Los Angeles, CA 90089, United States of America; 2 Division of Biokinesiology and Physical Therapy, University of Southern California, Los Angeles, CA 90089, United States of America

**Keywords:** biped, brain–body-task, co-adaptation, locomotion, motor-babbling, limited-experience, tendon-driven

## Abstract

Inspired by animals that co-adapt their brain and body to interact with the environment, we present a tendon-driven and over-actuated (i.e. *n* joint, *n*+1 actuators) bipedal robot that (i) exploits its backdrivable mechanical properties to manage body-environment interactions without explicit control, *and* (ii) uses a simple 3-layer neural network to learn to walk after only 2 min of ‘natural’ motor babbling (i.e. an exploration strategy that is compatible with leg and task dynamics; akin to childsplay). This brain–body collaboration first learns to produce feet cyclical movements ‘in air’ and, without further tuning, can produce locomotion when the biped is lowered to be in slight contact with the ground. In contrast, training with 2 min of ‘naïve’ motor babbling (i.e. an exploration strategy that ignores leg task dynamics), does not produce consistent cyclical movements ‘in air’, and produces erratic movements and no locomotion when in slight contact with the ground. When further lowering the biped and making the desired leg trajectories reach 1 cm below ground (causing the desired-vs-obtained trajectories error to be unavoidable), cyclical movements based on either natural or naïve babbling presented almost equally persistent trends, and locomotion emerged with naïve babbling. Therefore, we show how continual learning of walking in unforeseen circumstances can be driven by continual physical adaptation rooted in the backdrivable properties of the plant and enhanced by exploration strategies that exploit plant dynamics. Our studies also demonstrate that the bio-inspired co-design and co-adaptations of limbs and control strategies can produce locomotion without explicit control of trajectory errors.

## 1. Introduction

Active and explicit control of robotic bipedal locomotion poses multiple challenges, including: (i) hybrid dynamics that transition among single- and double-leg stances and aerial phases [1], *and* (ii) actuators with insufficient bandwidth to manage instantaneous impacts [2]. To address these challenges, studies that take inspiration from the musculature of organisms have incorporated mechanical components and architectures to reduce limb inertia by implementing cable (i.e. tendon) driven structures [3], and increase the use of passive limb properties to manage impacts [2, 4]. Furthermore, approaches like zero moment point (ZMP) enable balance during bipedal locomotion via quasi-static foot placements [5], as in the ASIMO low-impact robot [6], which is built and programmed in a way that avoiding impacts with the environment is one important design consideration. Theories like hybrid zero dynamics (HZD) have been developed where a reset map allows the system to go back to stable performance after the intrinsic impulse perturbations of ground interaction in dynamic behavior [1]. In the furthest extreme, there are robots whose own structure allows them to produce locomotion without feedback signals that inform a computing system about body-environment interaction. An example of this is seen in [7] where a bird-inspired robot uses feedforward control (no sensory feedback) to produce locomotion; to do so it exploits its anatomy, which enables the robot to change its mechanical function at the moment the feet touch ground. This change is based on a spring-tendon structure that allows the legs to transition from aerial to on-ground states, enabling the production of useful steps. Proof of principle comes from passive walkers that can produce useful movements without sensors and/or actuators [8, 9].

The influence that the physical properties of a robot have on data acquisition and learning is at the core of the studies presented in this paper. Physical adaptation of robots to environmental constraints and conditions is a research area that can potentially improve the performance of walking robots when subjected to unforeseen circumstances [10]. In [11] a legged robot is able to achieve locomotion in novel environments by exploiting its morphological adaptability, showing that changing the robot physical configuration as a result of robot-environment interaction simplifies the task of learning new control strategies to walk in unforeseen environments. Another important point to improve learning of walking and its performance is to have more efficient ways, based on plant properties, to acquire training data. In [12] it is explained how a model, to be able to accurately describe and predict the behavior of a system, needs to be trained with data samples spanning throughout the entire range of possible values such samples could have. With our experiments we show how a training strategy that exploits a robot’s physical properties, in contrast to an strategy that naïvely forces the robot to perform regardless of its properties, produces data that is more relevant to the task to solve.

We take inspiration from how many biological organisms learn locomotion on their own: by co-adapting their brain (in robots this would be the controller) with their bodies and tasks to perform. An example of an application of control-adaptation in legged robotics is the work performed in [13], where it is highlighted how legged locomotion can emerge based on a robot’s control adaptability. Such study is an example of how body-environment interaction, in conjunction with feedback-based adaptable control, can drive the emergence of efficient and adaptive obstacle negotiation (i.e. more stable: with less variation in joint angles, hence more energy efficient). For the case of our studies, to create a bipedal robot that implements a brain–body-task co-adaptation to learn locomotion, we combined three bio-inspired features:

(i) Tendon driven limbs that are over-actuated (i.e. 2 joints, 3 actuators). This feature can be compared to the musculoskeletal systems of vertebrates where muscles pull on tendons to move joints. As tendons can only pull, redundant actuation is required: more than one tendon per joint is needed for both positive and negative joint rotations. These systems are simultaneously underdetermined (i.e. more than one tendon tension combinations can achieve the same limb posture and net joint torque) and overdetermined (i.e. only one tendon excursion combination allows for a particular limb position) [14]. Tendon driven systems present advantages over traditional torque driven systems (which are easier to control since single actuators directly drive individual joints). One advantage is the reduced limb mass (lower limb inertia), since motors can be placed closer to the center of mass of the system and further from moving joints (e.g. knee). Another advantage of this anatomy is the versatility that emerges from having more actuators than joins, for example in the index finger multiple tendons (i.e. 7) drive three joints, enabling the emergence of a feasible force set that allows for force and torque generation in multiple directions, facilitating grasp [15]. In the case of legs, versatile locomotion (related to the ability to generate forces with the feet in multiple directions while exploiting pendulum dynamics) depends on the control of a redundant muscle anatomy where individual joints are driven by more than one muscle [16].
(ii) Backdrivable limbs that in our robot exist due to backdrivability of its DC motors (i.e. low gear ratio in the gearhead that drives the output shaft of its DC motors). This allows environmental mechanical perturbations (impacts and contact with the floor) to counteract motor rotation, allowing the robot to adapt to environmental physical constraints. In [17], a backdrivable robot can interact with its physical surroundings allowing mechanical inputs to the robot affect its behavior. In [18] it is explained and shown with an artificial knee joint, how inherent flexibility in actuators can exist due to backdrivability; allowing better interaction with the environment while generating large torque output.
(iii) Motor babbling compatible with leg and task dynamics, that allows brain–body collaboration through sparse physical actions (akin to childsplay [19, 20]), to heuristically learn to perform tasks [21–24]. In the rest of the paper we further develop this point.

We present a ‘Natural’ motor babbling strategy as an extension of G2P or ‘General to Particular’ model-agnostic algorithm [25] which enables bio-inspired learning of locomotion movements in tendon driven robotic limbs. This natural babbling strategy is an improvement of the naïve babbling strategy previously used by G2P. Data collected during both, natural and naïve babbling, are used to train a simple 3 -layer artificial neural network (ANN) which represents the inverse map from 6D limb kinematics (i.e. for our robot proximal and distal joint position, velocities, and accelerations) to 3D motor control sequences (i.e. three motors actuating the joints through tendons).

In [25], it is seen how a naïve babbling strategy causes aproximately 80% of the data generated to lie on edges of the configuration space, away from the area where the locomotion solutions lie. In contrast to this naïve strategy (that persistenly coactivates antagonist actuators, imposing movements that can conflict with leg dynamics), natural babbling resembles muscle mutual inhibition in living organisms [26, 27]. This promotes a more informative sensory feedback, compatible with the limb properties. In detail, when performing natural babbling, motor activations: (i) produce joint rotations away from their limits of rotation and (ii) follow a sinusoidal patterns instead of step functions (with a phase shift of 180 +−20 degrees for pairs of motors that act on the same joint, in other words two antagonist motors are not simultaneously activated with high activation values). As a result the leg joints are more homogeneously exposed to the region of the configuration space where locomotion patterns lie, promoting a higher success rate of learning of robotic locomotion (ratio of experiments where walking is learned to those where it is not learned).

The main contributions of this paper are:

(i) To show a clear case where data-efficient training driven by plant dynamics can produce higher success rate of learning of walking and
(ii) To show that the need for feedback signals to adapt to unforeseen physical constraints can be significantly reduced by leveraging limbs backdrivable properties.

While our studies are not designed to account for the balance part of locomotion, we demonstrate with a physical robot that useful cyclical movements can emerge from the adaptation of actions learned from limited experience enabled by backdrivable limbs that implicitly manage body-environment interactions. These learning and adaptability approaches could be complemented by the computation of balance parameters based on theories like ZMP [5] or HZD [1] which explicitly considers the transitions between locomotor contact states in bipeds that do not use gantries to locomote. One of the critical factors offered by Natural Babbling is its data efficiency achieved by exposing the biped’s leg joints to areas in the configuration space where locomotion patterns lie, which can serve as a baseline for the lifelong learning of walking. All in all, our study emulates the adaptive behavior of animals, where continual success of learned actions relies on useful brain–body-environment interaction [28, 29].

## 2. Methods

### 2.1. Robot characteristics

**Figure 1.**
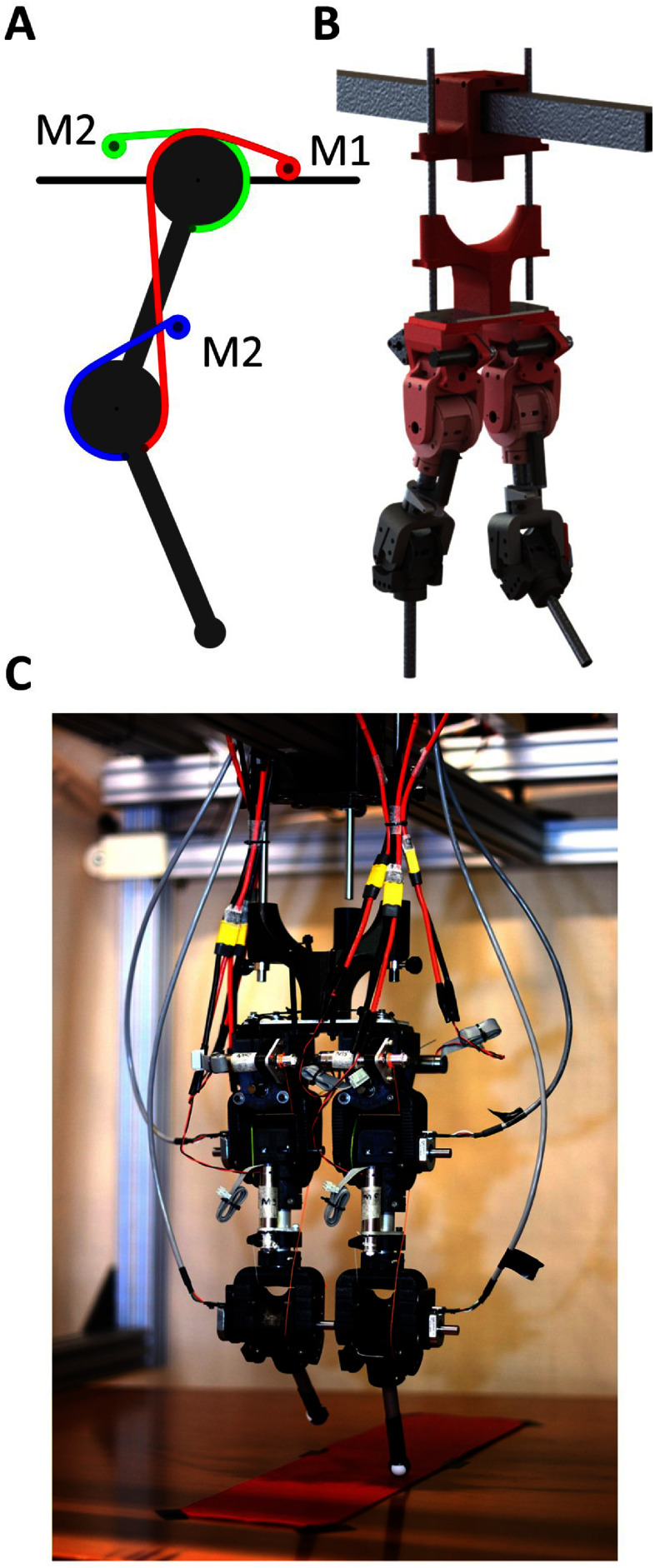
A Tendon route diagram of one leg, B Render of the 3D model of the biped C Photograph of the tendon-driven bipedal robot. To reduce rotational inertia, motors M1 and M2 (Maxon DCX16S GB KL 24 V, 21:1 reduction ratio gearhead) are placed distally to the joints.

For our experiments, we built and used a tendon-driven physical bipedal robot (figure 1). Each of its legs has hip and knee joints and a ball foot to facilitate the relative rotation of the lower section of the leg with respect to the ground.

The mechanical power to the joints is provided by a structure that resembles a muscle: the force is provided by a motor, while the muscle-joint interface (which in our robot would be the motor-joint interface) is a string that we call tendon. This robot is over-actuated since it has more actuators than degrees of freedom (DoF). The tendon route is shown in figure 1(a).

The tendon routing of our robot is an evolution of the routing for the robot in our already published paper [25], where all the motors were placed distally to the leg (i.e. in the hip). Here we simplify the tendon routing by having only two motors placed distal to the leg and one of them in the thigh. This design decision was made to reduce the torques driving the hip joint, thus potentially simplifying the task of learning a useful movement. The motors (Maxon DCX16S GB KL 24 V) include a gearhead (with a reduction ratio of 21:1). Respectively, each motor is called M1, M2, and M3, for details on their location please refer to figure 1. Comparing two motors A and B, both set to the same voltage level and mechanical load; A with a gearhead and B without one: motor A reduces the back-drivability of the limb while increasing its mechanical power output capabilities. This is an advantage for when the design of the robot is changed to a heavier one due to a bigger body size and/or the addition of more components (e.g. sensors and actuators).

The range of motion of the joints was bigger than for our previous robot designs, allowing us to explore the capability of the robot to track a desired trajectory independently of hard stops providing physical help. Here it is important to mention that in locomotion experiments the movement of a robot is typically physically limited by two components that serve as boundaries of its feasible configuration space: mechanical constraints (i.e. hard tops) in its own body, and environmental constraints (i.e, objects or ground itself). By designing our robot to have big ranges of motion normally not reachable while performing tasks (figures 4(b) and 5(b)), we focus on the role that environmental constraints have on the resultant performance of a task.

To maintain rotational inertia as low as possible (having a direct impact on power consumption to meet the demands of leg movement), and to increase the stiffness of the legs, we used aluminum tubes as main components of the legs. We used additive manufacturing or 3D printing techniques, for the construction of the joints. We also considered the implementation of easy tendon attachment points to facilitate the replacement of tendons, which is the part of the robot that breaks more often.

We built a gantry to support the biped, only allowing its hip to move along the *x* and *z* axis in its sagittal plane. The gantry prevents the biped from falling down, allowing us to focus merely on the task of learning a locomotion cycle.

### 2.2. General G2P overview

G2P stands for ‘General to Particular’. This is a bio-inspired algorithm which objective is to produce useful feedforward behavior in the physical world based on a data-driven approach. It uses motor babbling (random play) to explore and learn the ‘general’ capabilities of a physical system. Through motor babbling it creates a map from limb kinematics to corresponding motor activations. This map is then used to define ‘particular’ kinematic tasks to produce a desired outcome (an example of a desired outcome would be to generate locomotion cycles for walking). For this paper, the task that G2P generates is to follow a set of limb kinematics (a set of desired joint angular positions, velocities and accelerations) to follow a desired foot trajectory.

In the context of this paper, we refer to ‘naïve G2P’ as the G2P where motor babbling is driven by random ‘naïve’ motor activations; we refer to ‘natural G2P’ as the G2P where motor babbling is compatible with the dynamics of the legs. When referring only to the babbling strategies, we call them naïve and natural babbling depending on the used motor activation strategy. More details of both cases (i.e. naïve and natural G2P, as well as their respective babbling strategies) are explained in this and the next sections in Methods.

**Figure 2.**
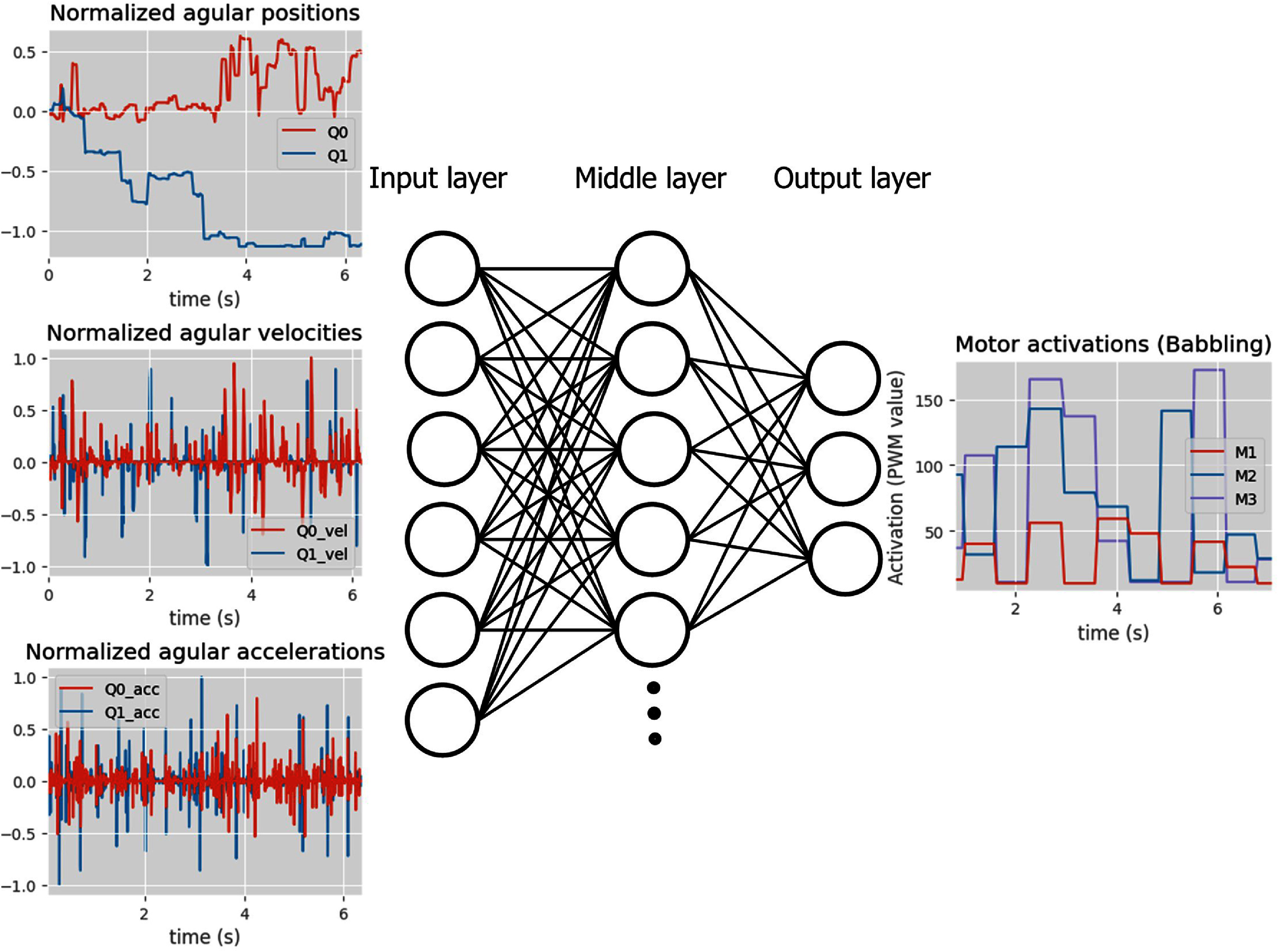
Representation of the ANN used as a map from six limb kinematics (input nodes, left column) to 3 motor activations (output nodes, right column). In this figure we show real data used to train the ANN (particularly naïve babbling data) As a reminder, motor activations in babbling are random (Specific details on naïve babbling are given in section 2.2). The ANN has three fully connected layers: input, hidden and output layers with respectively 6, 15 and 3 nodes. Note that motors are persistently simultaneously activated (i.e. coactivation, see motors M2 and M3 in leftmost panel), this decreases the spread in training data.

**Figure 3.**
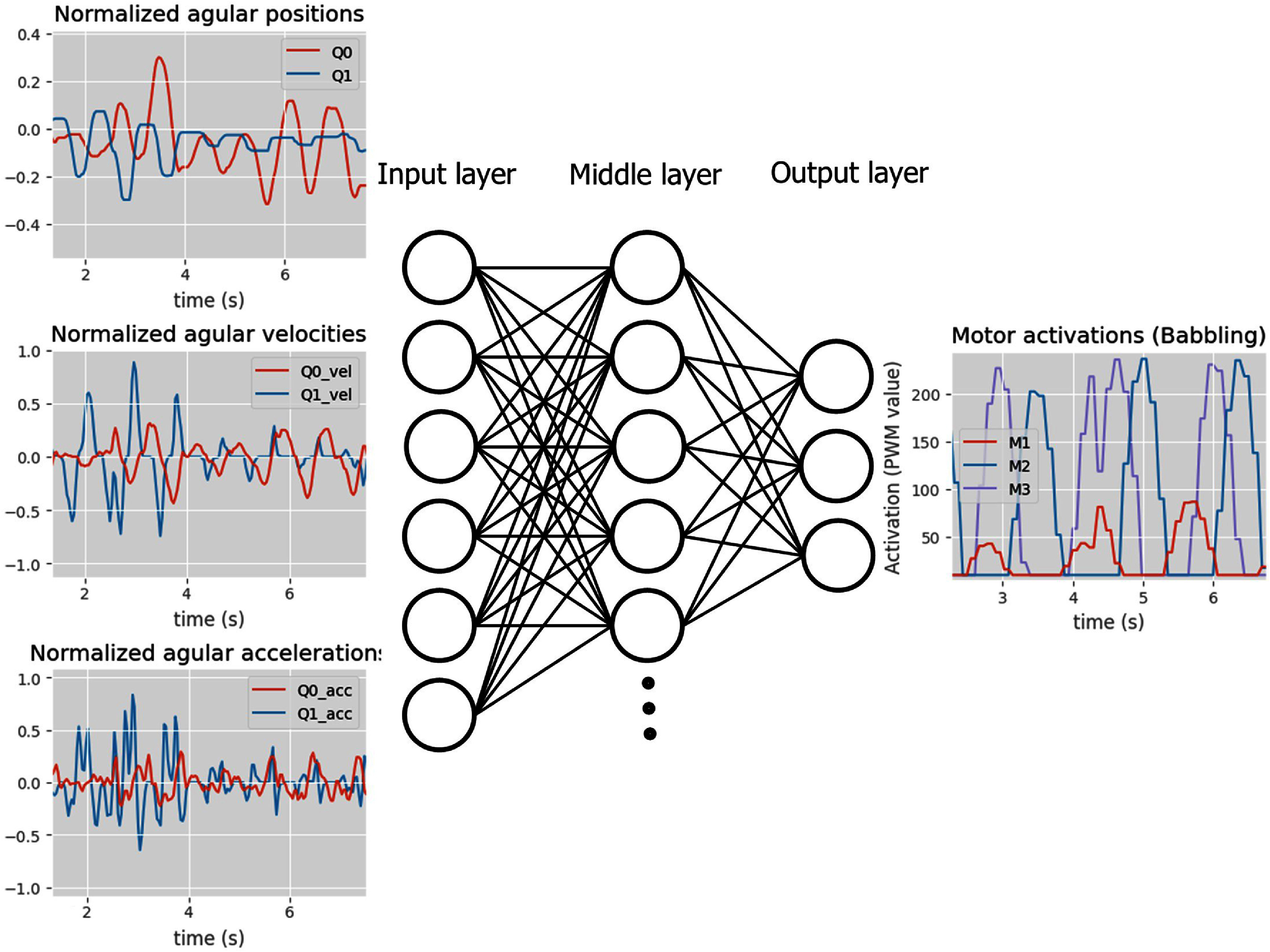
Representation of the ANN used as a map from six limb kinematics (input nodes, left column) to 3 motor activations (output nodes, right column). In this figure we show real data used to train the ANN (particularly natural babbling data) As a reminder, motor activations in babbling are random (Specific details on natural babbling are given in section 2.3). The ANN has three fully connected layers: input, hidden and output layers with respectively 6, 15 and 3 nodes. This figure shows how oscillatory movements are produced (see ‘Normalized angular positions’ panel) driven by the by oscillatory babbling activations (rightmost panel) with significant difference activation level between antagonist motors.

The first version of G2P was developed in [25]. This algorithm uses an ANN as a map from inputs to outputs (respectively desired kinematics to motor activations) (figures 2 and 3). The ANN is trained with input–output data sets obtained from babbling and tested with input-output data sets obtained from babbling by predicting outputs given inputs. The predicted outputs are compared with ground truth motor activations outputs. The difference between predicted and obtained values is the ‘training’ error. During babbling, the algorithm aims to reduce such training error. The testing/training data set size ratio is 0.25. We use one ANN per leg. In [25], G2P refines this map with a reinforcement learning approach, for this paper we do not consider such a section of the algorithm since we are interested in understanding the value of the data obtained during babbling.

The ANN used for Natural and naïve G2P (figures 2 and 3 respectively) represents the inverse map from 6D limb kinematics (i.e. for our robot proximal and distal joint position, velocities, and accelerations) to 3D motor control sequences (i.e. three motors actuating the joints through tendons), it has three fully connected layers (input, hidden and output layers) with 6, 15 and 3 nodes, respectively.

As the transfer functions for all nodes, we selected the hyperbolic tangent sigmoid function, which is an S-like function that produces a bounded output value in a range between −1 and 1. Additionally, we chose this function over the sigmoid since the gradient of the second is bigger than the first. The higher gradient produces a greater sensitivity to changes in the input values, producing higher updates in the weights of the networks (thus potentially faster learning). We also applied a scaling for the output layer (giving values between -1–1) to obtain values to cover the whole motor control range values (0–255).

The weights and biases were initialized based on the Nguyen-Widrow initialization algorithm [30, 31]; with this, we avoid initializing weights close to the regions where the gradient of the transfer function has very small or high values. Having initial values localized in the mentioned region creates undesired output saturation. To obtain the best results, this approach randomly initializes weights close to the midpoint of the transfer function (i.e. 0 for the cases of our experiments).

As a performance/error function, we used the mean square error (m.s.e.) approach. With this, the mean of the differences between values predicted by the ANN and the ground truth values are calculated. This loss function aims to minimize the overall training error.

This training error is propagated backward to update the initial weights, the action performed with the Levenberg–Marquardt back propagation technique, the assignment of new weights is particularly done with adaptive moment estimation (Adam), a gradient descent method chosen over MomeNtum, AdaGrad, RMSProp. Adam is the standard go-to method since it includes benefits from both Momentum and RMSProp. To find the best model weights, it leverages the usually seen speed of MomeNtum, and adaptability to gradients with different orientations commonly well handled by RMSProp. Each time the backpropagation is complete, it is considered that an epoch happened. We determined the maximum number of epochs to 100; also, the model training stops after there is no improvement after 5 epochs.

### 2.3. Natural babbling: changes to G2P babbling strategy

As mentioned in the introduction, we made changes to the babbling strategy of G2P to more homogeneously expose the leg joints to the areas in its configuration space where locomotion patterns lie. To keep our focus on assessing the usefulness of the data to produce a mapping with which a desired trajectory can be tracked, we particularly tested the G2P capability to create motor activations to limb kinematics map without any refinement to such a map. With this paper, we show that (for a two DoF, three actuators leg) properly obtained data can be enough to train an ANN to produce useful movement (more details in results and discussion sections).

Before explaining the details of natural babbling, it is important to highlight that naïve babbling consists of random step PWM signal variation for each one of the motors and that each motor signal is independent of the others (frequency of steps change: 1.3 Hz), as shown in figure 2, rightmost panel. For natural babbling, we modified the randomness of motor activations by including the rule that the activation level of two antagonist motors should be significantly different (As observed in motors (M) 2 and 3 in figure 3, rightmost panel).

**Figure 4.**
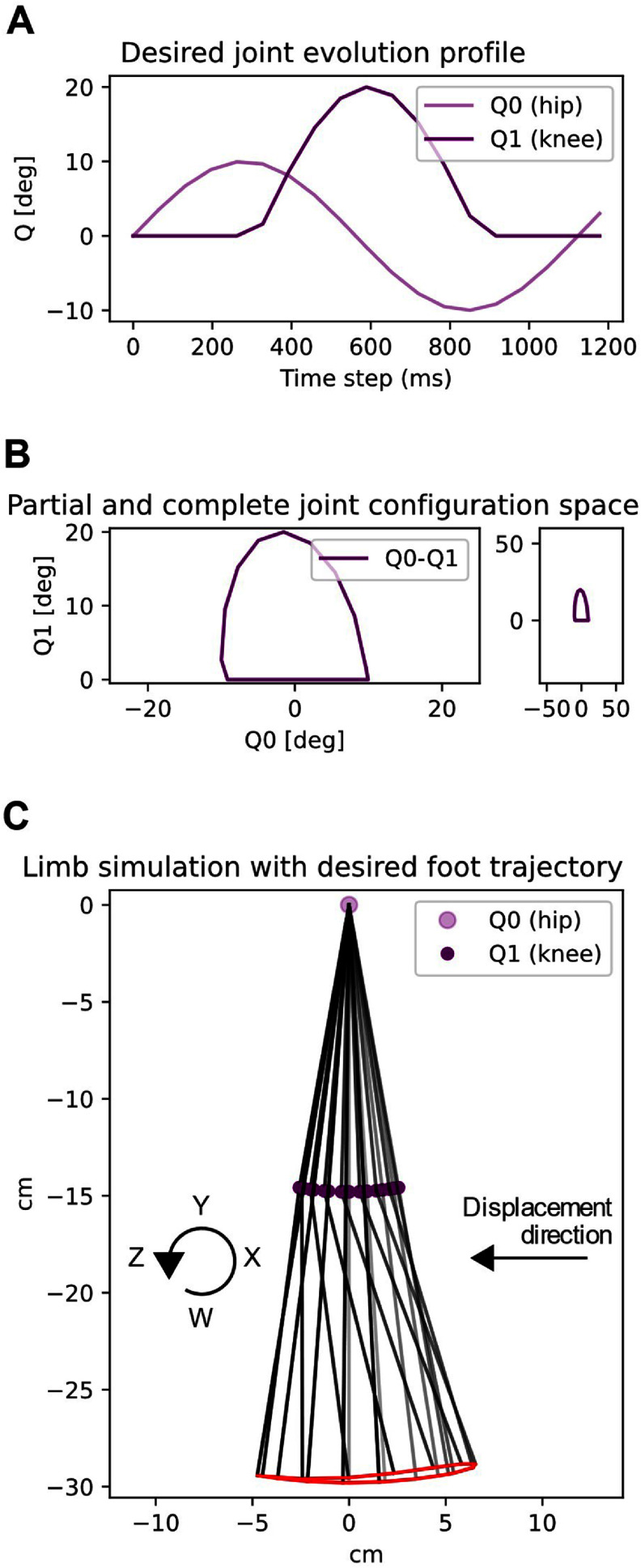
Desired joint and foot trajectories. In A it is shown a hip and knee joint evolution profile that produces limb movements away from the limits of its in-air configuration space as shown in B (Both panels in B represent the same, right one is a zoom out version). The resultant foot trajectory, shown in C is such that permits its front and back swings to have different height (necessary point to produce locomotion). Note that the desired foot trajectory is always kept at a constant distance from the hip; thus if the hip position is changed the desired foot trajectory will also change.

For natural babbling (figure 3), each PWM signal for each of the motors follows a sinusoid profile. Considering that the mean value of the signals is 0, only the positive section is used. For each motor, the signal amplitude is varied randomly. M1 and M2 signals have a phase shift of 180 deg. This is to avoid simultaneous activations of the motors which cause no hip movement to happen [26, 27]. Every 15 s, the phase between M1 and M3 was increased by 36 deg and the baseline of each signal varies +−30 PWM units (approximately +− 1 V). To get a sinusoid-like shape, steps in series need to be considered (this is a digital system, so we are discretizing the signal). Step frequency: 6 Hz. Sinusoid frequency (every time a period is completed):.6 Hz. Frequency of each signal peak: 1.3 Hz. Each peak (natural babbling) has approximately the same width as each step of naïve babbling. All frequencies are reported as approximate values. It is intrinsic to the microcontroller behavior to have slight variations in signaling and sampling frequency. The limits of rotation of each of the joints were never reached with natural babbling, a crucial point for our results and conclusions (figure 4(b)).

**Figure 5.**
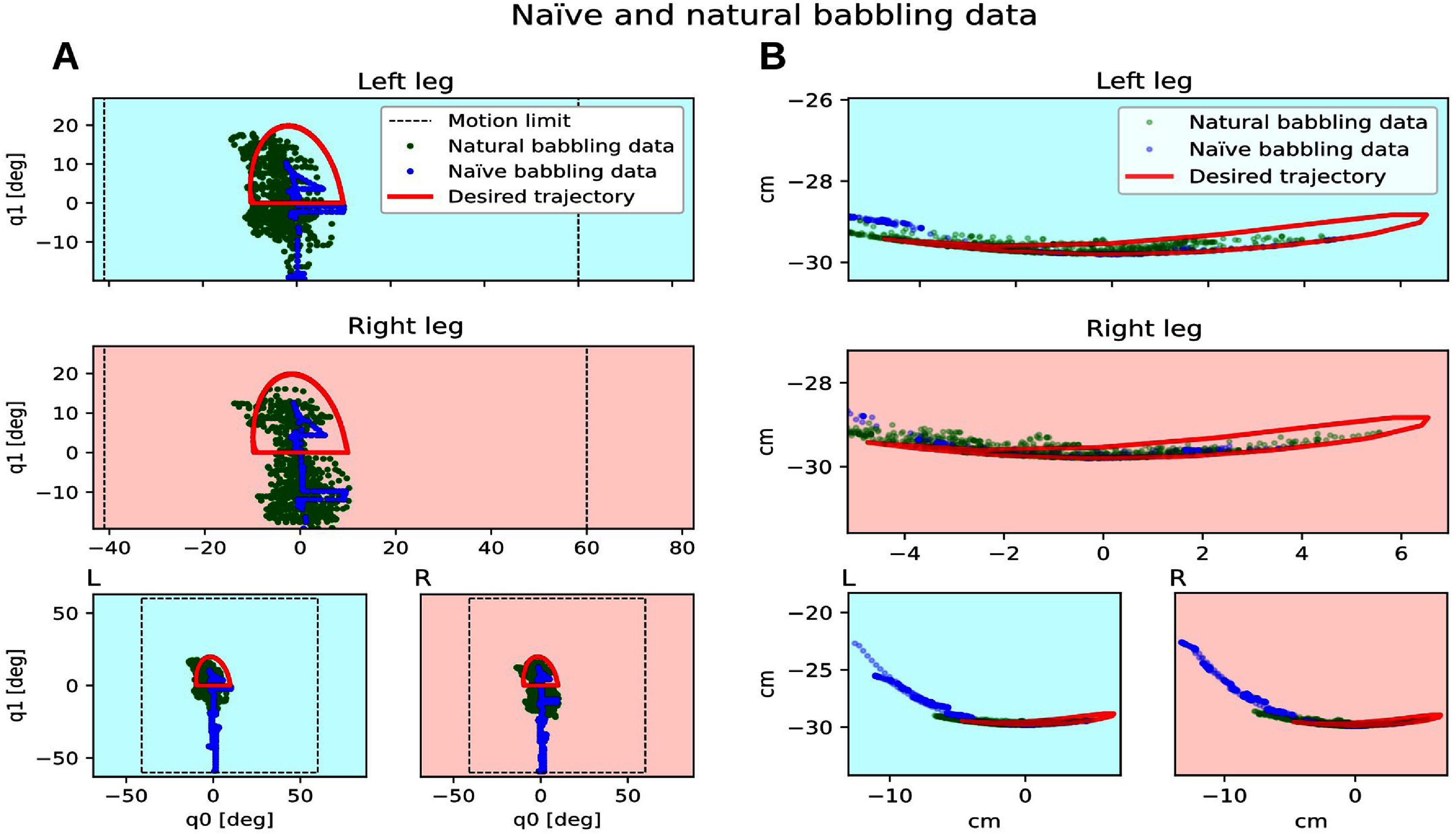
Two min of babbling data and desired trajectories for one trial. A: Joint Space with joint motion limits marked with a doted square, B: Endpoint Space. The spread values are 0.14 and 0.18 for naïve babbling data (left and right legs respectively) and 0.60 and 0.53 for natural babbling data (left and right legs respectively).

### 2.4. Desired foot trajectory characteristics and variations

Before hardware experiments were performed, we did a forward kinematics analysis of possible limb movements that allowed us to obtain the desired joint evolution profile and ranges shown in figures 4(a) and (b)). The resultant foot trajectory is such that allows its front and back swings to have different heights (figure 4(c)).

**Figure 6.**
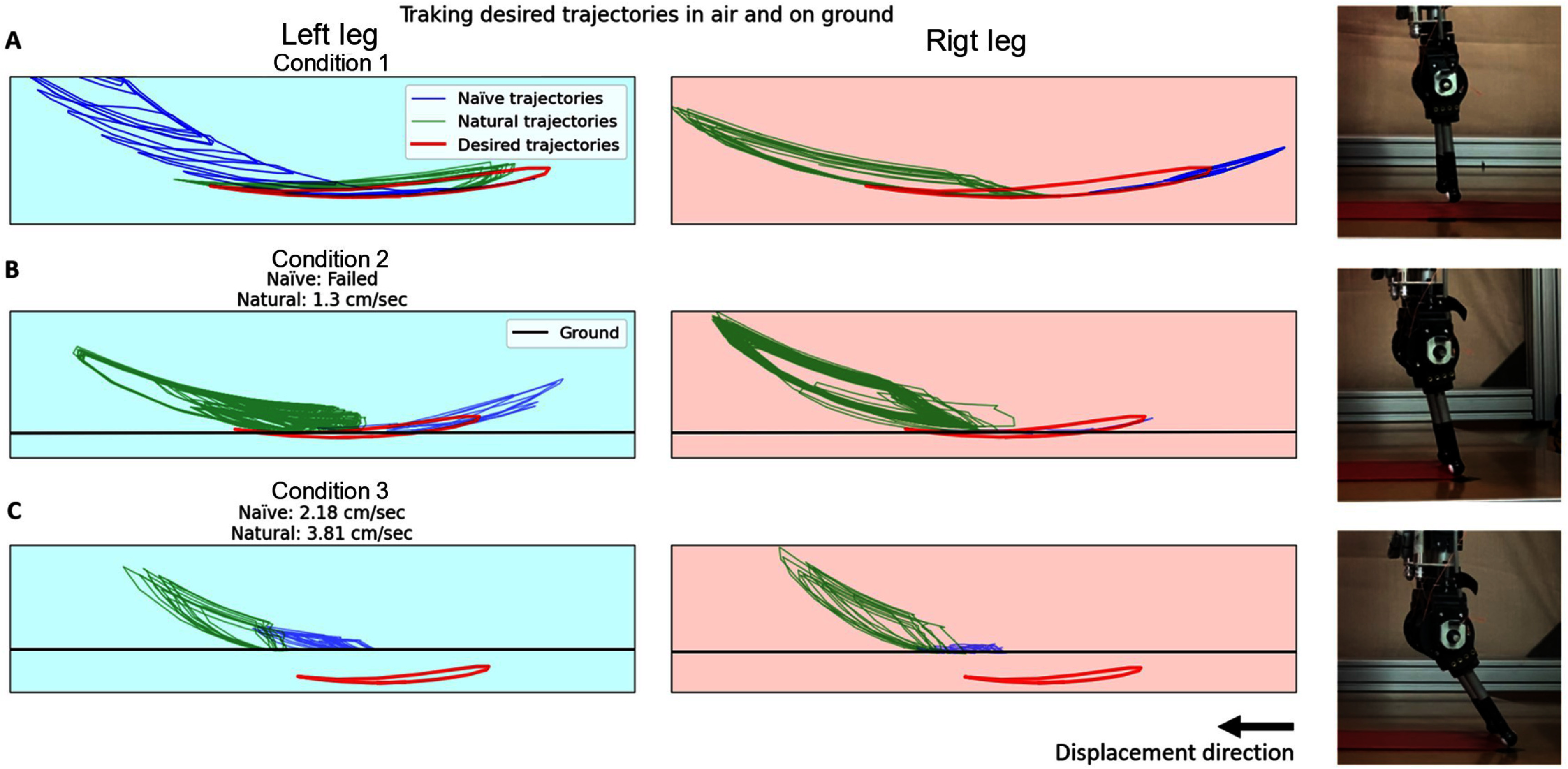
Plots of obtained and desired foot trajectories shown together with close ups of the biped feet in different conditions. ‘naïve and Natural trajectories’ means trajectories obtained from training with naïve and natural babbling (this figure corresponds to the same trial as the one presented in figure 5): Condition 1 (A): Desired trajecotries in air, Condition 2 (B): Desired trajectories in slight contact with the ground (locomotion emerges when training with natural but not with naïve babbling) and Condition 3 (C): Desired trajectories 1 cm under ground (locomotion emerges when training with both natural and naïve babbling).

As shown in figure 6, we divide our experiment into three main conditions determined by the location of the desired trajectory with respect to the ground. The desired trajectory always has the same distance to the robot’s hip, changing the position of the desired trajectory requires changing the robot’s hip height by re-configuring the gantry that prevents the robot from falling down (figure 1(c)). Depending on its location, a fraction or no part of the desired trajectory is reachable by the feet of the biped. We divide our experiments in three cases:

(i) **Condition 1: Desired trajectories in air-** only in air movement, with no interaction with the ground. When performing movements, the feet trajectories will be limited only by the characteristics of the biped itself (figure 6(a)).
(ii) **Condition 2: Desired trajectories in slight contact with the ground-** desired foot trajectories are only partially reachable since they are partially under the ground level. In other words, ground constraints the movement of the robot to stay over the boundary marked by the ground (figure 6(b)).
(iii) **Condition 3: Desired trajectories 1 cm under the ground-** desired trajectories are unreachable, they are completely under the ground level. This is the condition where the biped’s movements are more constrained. Also, for this condition, the area of the feasible joint configuration space is smaller than in points 1 or 2 (i.e. here the biped movements are constrained to exist between the limits imposed by the ground and the limits marked by the limits of joint rotations) (figure 6(c)).

### 2.5. Hardware experiments steps

The following steps were performed using both: naïve and natural babbling. Eight trials of this experiment were performed, four based on naïve babbling and four on natural babbling. If the biped displaces its body mass for 40 cm we consider this a successful walking trial. The success rate is calculated by dividing the number of successful trials by the number of performed trials of a particular kind (i.e. condition and type of babbling data used). When a result is reported as ‘mean’, it is the average value from four trials. For the mean cases of spread and detrended fluctuation analysis (DFA), the number of values considered is eight (left and right legs for each of four trials: total eight).

These are the steps we followed to perform our experiments:

(i) Collect babbling data for two min (figure 5). Babbling characteristics are described in section 2.2.
(ii) Train an ANN to map motor activations to limb kinematics as described in section 2.2.
(iii) With desired trajectories in air (i.e. section 2.4, biped suspended in air, no ground constraint), track the desired foot trajectory (figure 6(a)).
(iv) With desired trajectories in slight contact with the ground (i.e. section 2.4, biped’s hip at 40 cm off the ground). Perform trajectory tracking as in (figure 6(b)). Measure the time the biped takes to travel 40 cm in case there is successful walking.
(v) With desired trajectories 1 cm under the ground (i.e. section 2.4, biped’s hip at 39 cm off the ground, figure 6(c)). Measure the time the biped takes to travel 40 cm in case there is successful walking.

It is important to make the distinction between ‘training’ and ‘trajectory’ errors. Training error is the difference between obtained and predicted leg kinematics during babbling. Trajectory errors are the difference between desired and obtained trajectories while performing cyclical foot trajectory patterns, that error is neither modulated nor reduced.

### 2.6. Data analysis (Spread calculation)

We discretized the area within the desired trajectory into $1\times1\,\textrm{mm}^{2}$ pixels and checked if the foot visited that pixel during a single babbling trial. Then by calculating the ratio of the occupied pixels to all pixels, we quantified the spread. Spread quantifies how well the algorithm (specifically, the babbling) can explore different kinematics by knowing the locations that feet have passed through.

### 2.7. Data analysis (DFA)

In DFA, the fractal scaling component estimates a time series’ scaling behavior which represents the power law scaling behavior of the time series over various time scales. The steps for DFA are as follows:

(i) First, we detrended the time series data of the endpoint’s distance to the hip from each trial by dividing the time series into non-overlapping windows of equal length and then fitted a polynomial function of first degree to each window.
(ii) Then we divided the detrended series into smaller segments of equal length (boxes). The scale factor determines the length of the boxes.
(iii) Afterward, we calculated the root-mean-square fluctuation (F) for each box in the detrended series.
(iv) Then, we calculated the average root-mean-square fluctuations across all the boxes at a given scale.
(v) We repeated steps 1 to 4 for different scale factor values and plotted the average fluctuation versus the scale factor (DFA curve).
(vi) Finally, we analyzed the DFA curve to check the time series data for long-term correlations. The DFA curve shows a power-law relationship between the fluctuation and the scale factor quantified by the slope alpha (fractal scaling component) using linear regression on a log–log scale.

A higher fractal scaling component indicates that the time series exhibits stronger long-term correlations or persistence over various time scales, which means that the fluctuations in the time series at larger time scales are more correlated, and the time series has a more persistent trend. Conversely, for a lower fractal scaling component, this analysis indicates weaker long-term correlations or anti-persistence in the time series, which means that the fluctuations at larger time scales are less correlated, and the time series has a less persistent trend [32–35]. We use the persistence of trends and strength of correlation in the legs’ movements as a criterion to compare how well and robustly the biped walks (in case walking is achieved) in different cases and conditions.

## 3. Results

### 3.1. Exploiting limb mechanical properties increases the spread of training data and increases success rate of locomotion learning

All results reported in this subsection correspond to babbling data and walking attempts for Condition 2: Desired trajectories in slight contact with the ground (figure 6(b)). As a reminder, the success rate is calculated by dividing the number of successful trials by the number of performed trials of a particular kind (i.e. condition and type of babbling data used).

Two min of natural babbling data are enough to produce locomotion, while 2 min of naïve babbling data are not enough (figure 6(b)). With natural babbling G2P learned walking in 75% of the trials compared to 0% for naïve babbling trials. Mean displacement speed for successful natural-babbling-based trials was 1.9 cm s^−1^ speed for 3 out of 4 successful trials: 2.45, 1.96, 1.3 cm s^−1^.

The difference, as previously described, between the naïve and natural cases resides in the babbling data. More spread babbling data (i.e. natural babbling data are more spread compared to naïve babbling data, as shown in figure 5) shows that the babbling was more successful in exploring the leg kinematics, which is the primary purpose of babbling. Consequently, compared to natural cases, a lower success rate happen when training with naïve babbling data.

As shown in figure 5, natural babbling data are closer to the regions of the configuration space where locomotion solutions lie. If we analyze the spread of this data within the area delimited by a desired trajectory, we see that the spread for the natural babbling data is higher than that of the naïve babbling data. For the trial presented in figure 5, left–right leg spread of naïve babbling data: 0.14 and 0.18 respectively; left–right leg spread of natural babbling data: 0.60 and 0.53 respectively. Mean spread values for naïve and natural babbling data respectively are: 0.55 and 0.95.

In [12] it is described how a model to be able to describe a system, and to accurately predict its behavior, needs to be trained with more training samples spanning throughout the entire range of possible values such samples could possibly have. In our experiments most of the naïve babbling points lie away and few inside the desired trajectory, in many cases failing on training a model that can accurately predict the behavior inside the desired trajectory. In this work behavior will be the motor commands to pull on the tendons to produce cyclical movements that are close to the desired trajectory. This is seen in figure 6(a) where the blue trajectories based on a model trained with naïve babbling data fail to closely resemble the desired trajectory. In contrast natural babbling points, which are more spread and lie inside of the desired trajectory are better to train a model which can predict the motor activations required to produce cyclical foot trajectory patterns that better resemble the desired trajectory. This is seen in figure 6(a) where the green trajectories based on a model trained with natural babbling data better resemble the desired trajectory compared to the case of the naïve babbling based experiments.

### 3.2. Placing desired trajectories completely under ground level increases walking success rate and produces faster walking

When the desired trajectories were 1 cm under the ground (Condition 3) (figure 6(b)), G2P learned supported bipedal walking in 100% of the trials based on both naïve and natural babbling. naïve case speeds (1.79, 3.27, 1.7, 2.18 cm s^−1^), natural case speeds (5.03, 4.93, 6.19, 3.81 cm s^−1^) Respectively, mean displacement speeds for this cases were 2.23 cm s^−1^ and 4.99 cm s^−1^ For trials based on natural babbling, when going from the condition where the desired trajectories are in slight contact with the ground (Condition 2) to the condition where the desired trajectories are 1 cm under ground, mean speed increased by 262%, and success rate was increased from 75% to 100%. For the trials based on naive babbling the success rate was increased from 0% to 100%.

For the condition where the desired trajectories are in slight contact with the ground (Condition 2), the biped can only barely touch ground with fully straight legs, reducing the work that the legs produce to only the swing of the hip. In contrast, when the desired trajectories are 1 cm under ground (Condition 3), the biped can produce work with both hip swing and knee flexion (figure 6).

**Figure 7.**
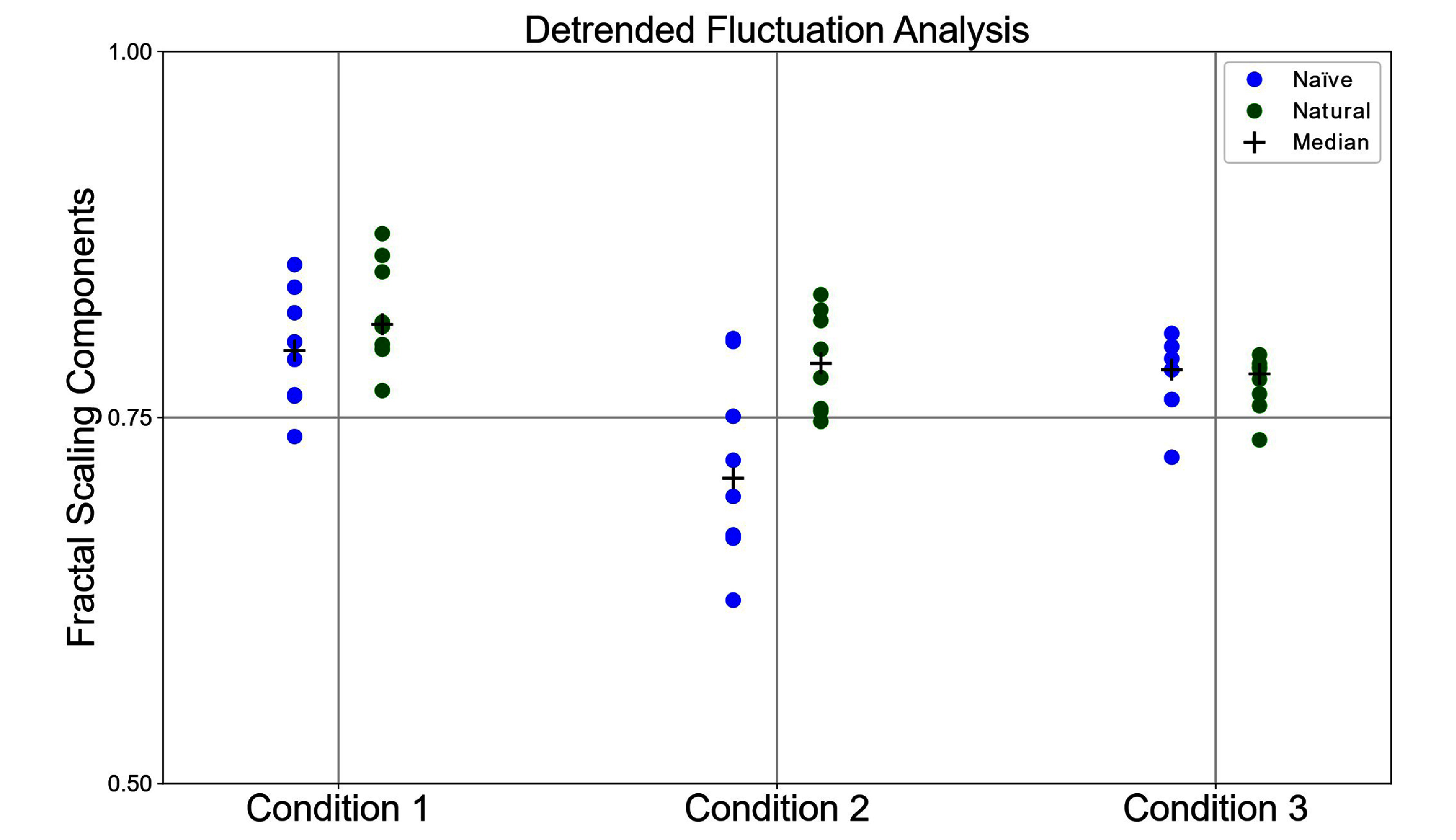
This figure shows the dot plot of the fractal scaling components for naïve (blue) and natural (green) cases from eight different trials (four trials from each leg). Conditions 1, 2, and 3 show fractal scaling components when the desired trajectory is in the air, in slight contact with the ground, and 1 cm under the ground, respectively.

Compared to the biped’s in-air performance (desired trajectories in air), when the desired trajectories make slight contact with the ground, the scaling behavior decreases (See DFA in Methods, section 2.7) across all our experiments (figure 7). This indicates, as expected, that tracking ground trajectories is more complex for the biped than tracking in-air trajectories. When trained with naïve babbling data and the desired trajectories are 1 cm below the ground, the resulting movement exhibits significantly higher scaling components (*p* ≈ 0.03) compared to when the desired trajectories slightly touch the ground, indicating more persistent locomotion. Conversely, when trained with natural babbling data under the same conditions, there is no significant difference in scaling components (*p* ≈ 0.22), though there is less variance between trials.

For cases trained with natural babbling data, when taking the desired trajectory from slightly touching ground to being completely under ground, there is an increment in walking speed. The reason for this is that, for cases based on natural babbling training data, walking has already emerged when the desired trajectory slightly touches ground. In the other hand, for cases based on naïve babbling training data, walking first emerges when the desired trajectories are placed 1 cm under ground. Both naïve and natural cases present an improvement when trajectories are placed under ground level, but naïve cases has less improvement after locomotion emerges than cases based on natural babbling.

## 4. Discussion

This paper aims to motivate the creation of bipedal robots that learn locomotion via data-driven co-adaptation with the dynamics of the plant to manage interactions with the environment. This is made possible by using motor babbling to inform a motion planning strategy that produces cyclical movements that can undergo useful adaptations thanks to the backdrivable and impact-resilient properties of the legs. These properties allow the unsupervised modification of a previously learned behavior to enable the emergence of locomotion under different (previously unseen) conditions. We find that a bio-inspired approach to ‘natural’ motor babbling compatible with the dynamics of the tendon-driven legs improves the success of locomotion learning and performance compared to ‘naïve’ arbitrary motor babbling. The techniques presented here could be further complemented by other relevant approaches such as the calculation of parameters useful to maintain a balanced gait such as ZMP [5], or HZD [1] that explicitly considers the transitions between locomotor contact states. Even though these techniques are not necessary for the successful performance of our robot, in general they are potential options to further complement the experiments of this paper which do not focus on balance, but particularly on the generation of useful cyclical movements for locomotion.

A central aspect of our results is that the robot’s backdrivable limbs interact with the environment by allowing their movements to adapt to where the desired trajectory of a walking action is located with respect to the ground: in air, in partial contact with the ground (partially reachable) or under ground level (unreachable). For each of these conditions, interference of the desired trajectory with the ground was progressively greater, and the adaptation of a previously learned action was automatically modulated. Thus, the success of the resulting behavior does not depend on explicitly modulating or reducing desired vs. obtained trajectory errors. Rather—similar to the adaptive behavior observed in the locomotion of insects [36], crustaceans [37], and birds [38]—successful locomotion emerges because of, and not in spite of, brain (or controller)-body-environment interactions [28]. This adaptation happened with a performance strategy not explicitly aware of interference or impacts with the environment.

For the natural babbling case (compared to naïve case), we found higher fractal scaling components for cyclical movements with ground interference, as shown in figure 7-condition 2, suggesting they are more persistent. In the case of naïve babbling, increased ground interference had a more profound effect. When there was slight contact with the ground, we saw no locomotion and lower fractal scaling components (figure 7-condition 2). But when contact with the ground was further increased (figure 7-condition 3), locomotion emerged from cyclical movements with higher fractal scaling components comparable to those for natural babbling. These results points to counterintuitive controller-body-environment interactions that produce better locomotion as interference with the ground increases. While we expected the reduction of the workspace of the leg to hamper locomotion, it seems the compliance of the legs (due to their backdriveability) adapt sufficiently well to shape the limits cycles to produce locomotion without the control signals being explicitly aware of control trajectory errors.

We refer to our natural babbling approach as a brain–body-task co-adaptation technique. This statements is based on:

(i) During babbling, the body postures and behavior are driven by motor activations, as shown in figures 2 and 3 where the leg kinematics (i.e. joints angular positions, velocity and accelerations) depend on the motor activations. In other words the body is adapting to brain inputs.
(ii) A map (represented by an ANN with specific weights and biases) of movement to motor activations is created (i.e. central part of figures 2 and 3). This map depends on the resultant limb kinematics produced by a particular train of commands dictated by natural or naïve babbling. For the particular case of natural babbling, there is a connection between the brain (ANN) and the plant properties. The plant, through its properties, significantly determines the ANN generated. In other words we say that the brain is adapting to the body’s properties.
(iii) Using the map generated in (ii), the limb is tasked to move with different kinematic profiles (i.e. joints positions, velocities and accelerations which depend on step (i) and (ii)) to reach an specific desired trajectory. In figure 6 it can be seen how different training strategies produce different kinematic profiles to reach a desired trajectory. Here we say that the task (to follow a particular kinematic profile to reach a desired trajectory) depends on (or adapts to) the body which behaves differently depending on the used babbling strategy.

When following a naïve babbling approach, the brain (ANN) is not adapting to the plant mechanical properties and dynamics (i.e. over-actuated, under- and over-determined tendon driven pendulum system; properties explained in the Introduction and Methods sections). Naïve babbling does not let the brain co-adapt with the properties of the body, but to a behavior driven by a naïve approach that forces the plant to perform actions regardless of its properties. In the other hand, when performing natural babbling, the brain adapts to the properties of the plant, producing a higher success rate of locomotion learning.

Even though we do not address the balance part of bipedal locomotion (which is a bipeds capability to stay in upright position regardless of the instability effect that gravity pull imposes on it), gravity still affects the plant behavior and thus the characteristics of the ANN (i.e. map from limb kinematics to corresponding motor activations). In other words, the brain (ANN) is affected by the body which behavior is related to gravity. Together with the physical characteristics of the plant (i.e. mass, inertia, dimensions, etc), gravity determines the swinging properties of the legs. Our robot learns to coordinate antagonistic actuators to move a limb which pendulum-like behavior significantly depends on gravity (i.e. when the leg segments are not perpendicular to ground, gravity accelerates or deaccelerates their movement depending on their instantaneous swinging direction; this gives rise to the pendulum’s oscillatory nature).

As mentioned before, one fundamental aspect of this study is that we prescribed a type of motor babbling (i.e. natural motor babbling) that is compatible with, and exploits the bio-inspired mechanical properties of the tendon-driven limbs. Although similar in principle to Berniker *et al* [39], where the anatomical properties of a bio-inspired limb are exploited, we develop these strategies directly in hardware (and not in simulation). Moreover, we do not explicitly simplify the task by prescribing recurring muscle patterns (i.e. muscle synergies) to produce limb movements. It is our natural motor babbling that implicitly finds useful patterns of motor activations to the tendons. In fact, our natural motor babbling is one of the important extensions to out prior work on autonomous learning of locomotion [25]. By using this type of motor babbling that tends to avoid antagonist motor commands, we take inspiration from biological organisms where co-contraction can be energetically wasteful. While co-contraction can be considered as energetically wasteful, it can also be a means to control limb impedance and other physical constraints in animals, so it can also be a feature [40, 41]. Actions that leverage the backdrivable mechanical properties of the plant, compatible with the over-and under-determined actuation of its tendon-driven limbs, are parallel to one of the fundamental blocks of limb function [14, 29] to produce oscillatory limb movements (e.g. leg swing [27]).

## 5. Conclusion

We made changes to the training babbling strategy of G2P to more homogeneously expose a biped’s leg joints to the areas in its configuration space where locomotion patterns lie. We did that by implementing a natural babbling strategy that exploits the tendon-driven bio-inspired mechanical properties of its limbs (i.e. oscillatory movements produced by oscillatory activations, with significant difference activation level between antagonist motors). We observed that natural babbling reduces the spread of training data and increases the success rate of locomotion learning when environmental constraints are minimal (Condition 2 of our experiments). Furthermore we also observed that increasing environmental constraint to the system (interference between ground and desired trajectories) increased the tendency of the plant to behave homogeneously between different trials (regardless of trials being based on natural or naïve babbling). This shows how, even though the environment (i.e. ground) generates a higher desired vs. obtained trajectory error, it also collaborates with the backdrivable biped legs by ‘guiding’ them to perform a successful task by reducing their feasible configuration space.

We present proof-of-principle that effective locomotion can emerge from brain–body-environment interactions driven by a controller that does not aim to reduce errors with respect to desired locomotion trajectories. We find that these effective interactions arise from the co-adaptation facilitated by bio-inspired backdrivable properties of limbs. Moreover, the cyclical movements motor commands are informed by pseudo-random motor babbling that exploits and leverages the bio-inspired tendon-driven mechanical and dynamical properties of the limbs. This demonstrates the bio-inspired co-design and co-adaptations of limbs and control strategies can produce locomotion without explicit control of trajectory errors.

## Data Availability

The data that support the findings of this study will be openly available following an embargo at the following URL/DOI: https://github.com/DarioUrbina/natural_babbling Data will become available on: 29 November 2024.
